# Placenta accreta spectrum: From predictable risk to preventable crisis

**DOI:** 10.1002/pmf2.70259

**Published:** 2026-02-27

**Authors:** Bo Jacobsson, Teresia Svanvik

**Affiliations:** ^1^ Department of Obstetrics and Gynecology, Institute of Clinical Sciences, Sahlgrenska Academy University of Gothenburg Gothenburg Sweden; ^2^ Department of Obstetrics and Gynecology Sahlgrenska University Hospital, Region Västra Götaland Gothenburg Sweden; ^3^ Department of Genetics and Bioinformatics, Domain of Health Data and Digitalisation Institute of Public Health Oslo Norway; ^4^ Department of Obstetrics and Gynecology Södra Älvsborgs Hospital, Region Västra Götaland Borås Sweden

Placenta accreta spectrum (PAS) is a life‐threatening complication of pregnancy whose incidence continues to rise. This increase is not unexpected, but rather a predictable consequence of contemporary obstetric practice. Escalating cesarean delivery rates has fundamentally reshaped the obstetric risk landscape for PAS. A history of cesarean delivery confers substantial risk, which rises sharply with each subsequent operation. This risk is further amplified by other forms of uterine injury, including myomectomy, dilation and curettage, uterine irradiation, endometrial ablation, and hysteroscopic lysis of adhesions for Asherman's syndrome. The incidence of PAS has increased in the United States by a factor of 8 since the 1970s and in some settings, PAS now affects as many as 2–3 per 1000 pregnancies [1, 2]. These surgical risk factors, compounded by expanding use of assisted reproductive technologies and demographic shifts toward delayed childbearing, have driven a steady rise in PAS incidence [1]. Many of these surgical interventions are, in individual cases, entirely appropriate and can be lifesaving and contribute to improved maternal and perinatal outcomes. However, this is not universally the case. A critical and often overlooked opportunity to reduce the future burden of PAS lies in preventing unnecessary uterine surgery, particularly avoidable primary and repeat cesarean deliveries, thereby addressing PAS risk at its source.

The dangers of PAS are well known and well documented. Massive hemorrhage, emergency peripartum hysterectomy, and severe maternal morbidity are defining features of this condition. Historical data show that prior to protocolized screening, half to two‐thirds of PAS cases were unsuspected before delivery. What remains difficult to justify is how often PAS is still unsuspected at the time of delivery, specifically in the setting of placenta previa and anterior low‐laying placenta with a previous cesarean delivery. Each missed suspicion represents a patient facing hemorrhage in an unprepared setting, a surgical team without adequate resources, and a health system forced into crisis response rather than planned care. This persistent failure is not due to insufficient evidence or inadequate imaging tools. On the contrary, the obstetric and imaging communities possess robust data showing that PAS can be detected antenatally using ultrasound techniques that are widely available and readily incorporated into routine care. Early suspicion enables referral, delivery planning, and multidisciplinary management, interventions proven to reduce maternal and neonatal risk. Yet PAS continues to rank among the most unanticipated life‐threatening emergencies in obstetrics. This gap between knowledge and practice reflects not a failure of technology, but a failure of health systems, standards, and urgency.

In this issue of *Pregnancy*, an expert panel convened by the Pan‐American Society for the Placenta Accreta Spectrum confronts this gap [3]. By reviewing existing evidence, risk stratification models, and prior consensus statements, the panel provides clear, practical recommendations for antenatal PAS screening. Their central message is both simple and powerful: all patients with placenta previa or an anterior low‐lying placenta and a history of cesarean delivery should undergo targeted PAS screening at the time of the mid‐trimester anatomic survey [3]. Early, systematic assessment and timely referral when concern arises must become standard practice.

The rationale for this recommendation is compelling. PAS risk increases dramatically with the number of prior cesarean deliveries, particularly in the presence of anterior placenta previa or anterior low‐lying placenta. Additional uterine surgery further compounds the risk. In these high‐risk populations, combined transabdominal and transvaginal ultrasound with grayscale and low‐flow color Doppler reliably identifies characteristic markers of PAS, including loss of the clear zone, myometrial thinning, placental bulge, bladder‐wall interruption, uterovesical hypervascularity, placental lacunae, and bridging vessels. When standardized imaging protocols and structured reporting are implemented, prenatal detection improves dramatically, approaching rates as high as 97% in coordinated referral networks [4, 5].

Crucially, this problem is not confined to tertiary referral centers. In fact, the consequences of unsuspected PAS are often most severe in small delivery units, rural hospitals, and facilities where obstetric coverage relies on on‐call teams with limited subspecialty support. In these settings, unexpected PAS can rapidly overwhelm available resources, placing patients and clinicians at extraordinary risk. For such hospitals, antenatal screening and referral are not optional enhancements, but are essential patient safety strategies. A standardized screening protocol empowers frontline clinicians to recognize risk early and to transfer care before an emergency unfolds, rather than managing catastrophic bleeding in isolation at night or on the weekend.

Effective PAS screening depends on health system strengthening across multiple levels. Establishing a patient's a priori risk requires coordinated communication between referring clinicians, sonographers, radiologists, and obstetric and midwife providers. Ultrasound referral forms must consistently capture prior cesarean history and uterine surgery, a primary risk factor identification. Imaging protocols must be standardized, not left to individual discretion: a secondary ultrasound examination with possible follow‐up in late second trimester or early third trimester. Reporting must be structured to clearly convey concern and trigger referral pathways [3]. Without these system‐level supports, even skilled clinicians will miss opportunities for early suspicion.

Patient‐related barriers further complicate this landscape. Language barriers, limited health literacy, transportation challenges, and lack of trust in healthcare systems can delay recognition, evaluation, and timely referral. Addressing PAS, therefore, requires not only technical excellence but also patient engagement and equity‐focused care delivery. A culture of shared responsibility among providers, institutions, and patients is essential to closing these gaps. In this context, international federation of gynecology and obstetrics (FIGO) has developed the FIGO Pregnancy Passport, a standardized tool designed to ensure that critical obstetric information, such as prior cesarean delivery and uterine surgery, is consistently documented and accessible across care settings [6]. By enabling women to carry this information with them, the Pregnancy Passport has the potential to both strengthen health systems while simultaneously empowering patients to participate actively in risk recognition and safe care planning.

Ultimately, PAS exposes the true resilience of obstetric care systems. When risk factors are known, imaging markers are validated, and effective interventions exist, preventable harm becomes a systems failure rather than an unavoidable complication. The recommendations put forward by this expert panel represent an achievable first step toward standardizing care. They do not call for new technology or specialized imaging everywhere, but for consistent application of what is already known, particularly in the settings where failure carries the greatest cost.

For rural and low‐resource settings, the implications of this consensus are especially urgent. These facilities are often the least equipped to manage the sudden hemorrhage, prolonged surgery, and multidisciplinary demands imposed by unsuspected PAS, yet they are frequently where such emergencies unfold, particularly during nights, weekends, or periods of limited staffing. In these contexts, antenatal PAS screening is not an added burden but a critical risk‐reduction strategy. A simple, standardized ultrasound assessment performed at the mid‐trimester anatomic survey, coupled with clear referral pathways, can mean the difference between a controlled transfer of care and a catastrophic, unplanned emergency. Strengthening health systems to support early suspicion and referral of PAS in rural and low‐resource settings is therefore not a matter of specialization, but of equity, ensuring that geography, staffing models, or resource limitations do not determine maternal outcomes.

In summary, the authors emphasize the importance of targeted placental evaluation for PAS at the time of the mid‐trimester anatomic survey in patients with a history of cesarean delivery and anterior placenta previa or anterior low‐lying placenta as a first step toward standardized screening, acknowledging that this population represents the majority, though not all, cases [3]. Continued vigilance is also warranted in patients with additional risk factors for PAS. Timely referral of individuals with suspected PAS to centers experienced in its management is critical for optimizing maternal and perinatal outcomes, while also reducing the burden on clinicians faced with identifying and managing PAS in acute and often resource‐limited delivery settings.

If PAS continues to be identified only at delivery, it will not be because we lacked the tools to suspect it earlier. It will be because we failed to organize our systems to use them. Antenatal PAS screening is no longer a matter of clinical preference; it is a measure of health system maturity and a moral imperative in modern obstetric care. In this editorial, we endorse the authors’ message and frame it as a clear “call‐to‐action” for clinical implementation and health system strengthening.

## CONFLICT OF INTEREST STATEMENT

The authors declare no conflicts of interest.

## Data Availability

Data sharing not applicable to this article as no datasets were generated or analysed during the current study.
